# Ultrametric Factor Analysis for Building Hierarchies of Reliable and Unidimensional Latent Concepts

**DOI:** 10.1017/psy.2025.6

**Published:** 2025-03-06

**Authors:** Mariaelena Bottazzi Schenone, Carlo Cavicchia, Maurizio Vichi, Giorgia Zaccaria

**Affiliations:** 1 Department of Statistical Sciences, Sapienza University of Rome, Rome, Italy; 2 Econometric Institute, Erasmus University Rotterdam, Rotterdam, The Netherlands; 3 Department of Statistics and Quantitative Methods, University of Milano-Bicocca, Milan, Italy

**Keywords:** hierarchical factor analysis, higher-order models, latent variables (LVs), ultrametric correlation matrix

## Abstract

This article introduces a novel methodology to model the hierarchical dependence structure of manifest variables (MVs). This is done by reconstructing their correlation matrix considering a hierarchy of latent factors which forms an ultrametric correlation matrix. The proposed ultrametric factor analysis model will be shown to obtain reliable, unidimensional, and unique hierarchical factors. This approach addresses the limitations of traditional factor analysis methods that often oversimplify the multidimensional and complex relationships among MVs. The article provides an in-depth mathematical description of the proposed model, as well as an algorithm for parameter estimation. An extensive simulation study with 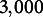 generated samples validates the proposal across twelve different scenarios. Finally, we demonstrate the potential of the proposed model using a real data set that is a benchmark in psychological research.

## 1 Introduction

Latent concepts represent aspects of reality that cannot be directly observed, such as quality of life, sustainable development, poverty, happiness, and personality traits. These concepts can be inferred through observable Manifest Variables (MVs) and are essentially measurable by means of a Latent Variable (LV) that is statistically related with two or more MVs. In general, latent concepts need statistical models to comprehensively and synthetically represent them through a mathematical formalization of the observed data and their relationships, i.e., the measurement model (Bollen & Bauldry, 2011, among others). However, the representation of a multidimensional latent concept through a single LV might result in a simplistic portrayal, by inadequately capturing the intricate nature of the complex phenomenon at hand. Exploratory Factor Analysis (EFA), pioneered by Spearman (1904), is one of the widely used statistical methods to identify LVs, often called *factors*, which are assumed to generate MVs and reflect into their linear relationships. EFA has been extensively applied to identify complex constructs like personality traits, intelligence, attitudes, and mental health in psychology; social stratification, cultural values, and interpersonal relationships in sociology; consumer preferences, brand perceptions, and product attributes in marketing; and quality of life, health behaviors, and disease risk factors in health sciences. Additionally, if a theoretical factorial structure can be hypothesized a priori, Confirmatory Factor Analysis (CFA), and more generally Structural Equation Model (SEM), can be used to test its fit to the observed data (Brown, 2015). Nonetheless, it may sometimes happen that a reduced set of factors, which are uncorrelated in EFA, is insufficient to analyze and reproduce the complexity of the phenomenon under study, since hierarchical relationships among its multiple factors exist.

Advanced approaches for exploring hierarchies of LVs underlying the data include hierarchical models, which organize various facets of a construct into hierarchical structures, extending from the original MVs to the ultimate General Factor (GF) through a refined group of Specific Factors (SFs). The GF represents the highest abstraction of the phenomenon under study, while the SFs denote dimensions that, in turn, measure specific concepts describing the primary components of the phenomenon under study. SFs are LVs less abstract than GFs and are more correlated with MVs, allowing a better understanding of the phenomenon. This hierarchical approach is necessary to provide a more precise and comprehensive understanding, steering away from the pitfalls of oversimplification associated with a single factor or with a group of uncorrelated factors. Higher-order factor analysis (HOFA; Gorsuch, 1983), second-order disjoint factor analysis (DFA; (Cavicchia & Vichi, 2022)), both based on EFA, and hierarchical disjoint principal component analysis (Cavicchia et al., 2023), a hierarchical extension of the model introduced by Ferrara et al. (2016), are examples of hierarchical models. Specifically, HOFA involves a sequential application of EFA followed by non-orthogonal rotation to detect higher-level factors representing broader constructs, while the other two models use a simultaneous approach, either limited to two orders or related to principal component analysis instead of EFA, respectively. In psychometric studies, hierarchical concepts are ubiquitous. For example, as described by Cattell (1971) and Carroll (1993) in the context of intelligence studies, a hierarchical model might include multiple levels of abstraction, with lower levels representing specific abilities or tasks (e.g., memory, verbal comprehension, spatial reasoning) and higher levels representing broader factors (e.g., fluid intelligence, crystallized intelligence, general intelligence or *g* factor). Another example is the Five-Factor Model of personality (Costa & McCrae, 1992; Cronbach, 1990), which organizes personality traits into five factors, namely openness to experience, conscientiousness, extraversion, agreeableness, and neuroticism, each represented by specific aspects. The aggregation of neuroticism, agreeableness, and conscientiousness on the one hand, and extraversion and openness to experience on the other, give rise to two higher-order dimensions, called alpha and beta (or stability and plasticity), respectively (DeYoung et al., 2002). Hierarchical models are also employed in organizational psychology to understand the structure of job performance (Campbell, 1990).

Unlike the aforementioned models, the methodology introduced in this article addresses the modeling of the unexplained correlation between higher-order factors through a simultaneous approach that goes beyond two levels and identifies higher-order LVs from the hierarchical structure of MVs. The proposal is called Ultrametric Factor Analysis (UFA), as it extends the EFA model to the case of factors with a hierarchical structure, which mathematically corresponds to an ultrametric correlation matrix. However, if UFA is applied to data without a true hierarchical structure, first-order factor correlations will be close to zero, resulting in near-zero loadings for higher-order factors. This indicates the absence of a hierarchical structure. The model requires that the identified factors possess certain relevant properties to define a sound methodology that adheres to the principles of high-quality statistics, as clearly described in the European Code of Practice (European Commission, 2017). Good properties for a hierarchy of factors include internal consistency, reliability, unidimensionality, and content validity of the factors. The article proposes an adequate measurement model for constructing a hierarchy of LVs with these desirable properties starting from a group of MVs. The results of the simulation study underscore the superiority of the UFA method compared to well-established factor analysis techniques, particularly in scenarios where the data exhibit a nested or hierarchical structure within factors. Traditional methods often struggle to accurately capture the complexity of such hierarchical data, leading to sub-optimal factor solutions. In contrast, UFA demonstrated a remarkable ability to identify and model the nested relationships, providing more precise and interpretable factor structures. We also apply UFA to the Cattell’s dataset, which focuses on the factorial study of intelligence. Given the inherent hierarchical nature of this construct, where specific cognitive abilities feed into broader dimensions like crystallized and fluid intelligence, UFA is perfectly suited to model this type of data. Indeed, by capturing both the unidimensional and hierarchical relationships by means of an ultrametric correlation structure, UFA provides deep insights into how different cognitive abilities interrelate, offering a more nuanced understanding of intelligence that traditional factor analysis methods may fail to capture.

The rest of the article is organized as follows. In Section 2, we present the notation used across all sections, along with fundamental concepts regarding ultrametric matrices, EFA and its constrained version based upon a sparse structure of the loading matrix. A comprehensive illustration of the proposed methodology is presented in Section 3. Section 4 describes the algorithm built to implement the proposed procedure. Different measures for model selection and for the choice of the number of factors are examined in Section 5. An extended simulation study designed to evaluate the model is illustrated in Section 6, while a practical application is the core of Section 7. Conclusions and key findings are reported in Section 8.

## 2 Notation and background

For the convenience of the reader, the notation and terminology common to all sections is listed here.

**Figure:**
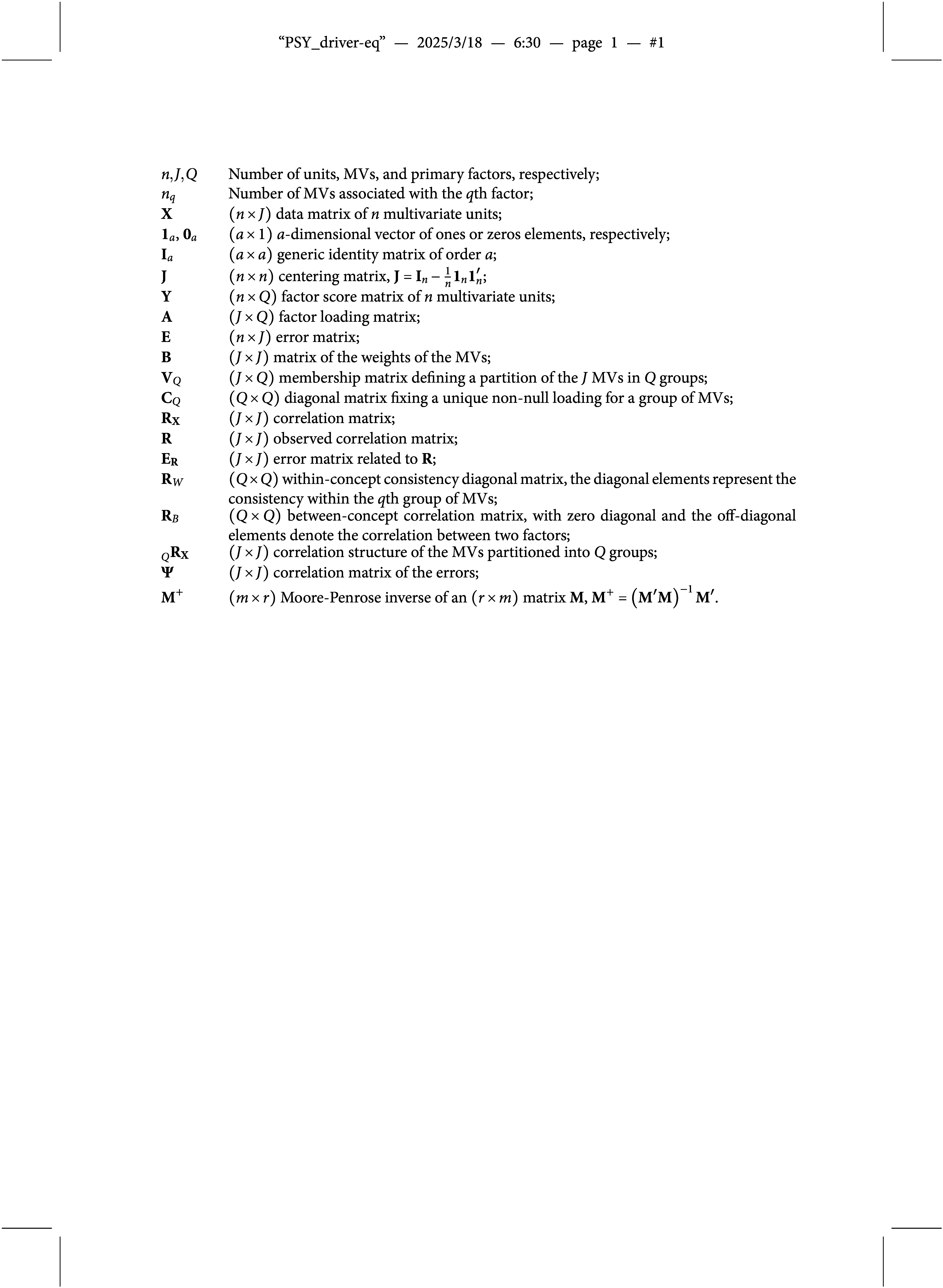

### 2.1 Ultrametric correlation matrix

To introduce our new factorial methodology, we first need to recall the definition of an ultrametric correlation matrix.

Definition 1. A matrix 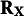 is an ultrametric correlation matrix if all its elements 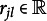 , for 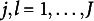 , satisfy the following conditions:

- symmetry: 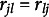 for 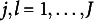 ;
- non-negativity and unitary of the diagonal: 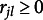 for all 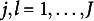 , 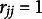 for all 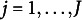 ;
- positive semi-definiteness (psd): 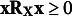 for all 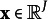 ;
- ultrametricity: 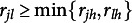 , for 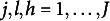 or, equivalently, for each triplet 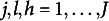 , there exists a reordering 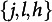 of the elements s.t. 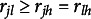 .

**Figure 1:**
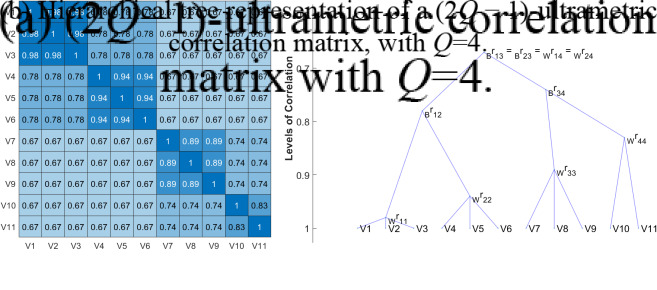
Example of a 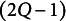 -ultrametric correlation matrix, with *Q*=4, and its corresponding hierarchical tree. First-order groups in (b) are 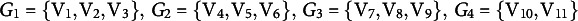 . Higher-order groups are obtained as 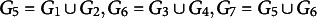 .

In detail, 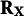 is a 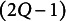 -ultrametric correlation matrix of order *J* if MVs are partitioned into *Q* groups and then they are agglomerated into 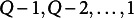 broader groups (Cavicchia et al., 2020). An example is provided in Figure 1a. Each group 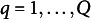 in the initial partition of the MVs is characterized by a strong correlation among the MVs within the group. This correlation level, denoted by 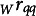, can be stored as the *q*th element of a diagonal matrix 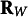 . The groups are also correlated with each other, though to a lesser extent. These correlations, denoted by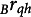, can be stored as off-diagonal elements of a hollow matrix 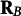 . To arrange the MVs into groups, and those groups into larger groups (property (iii) for the whole matrix), the correlations within each group must be greater than the correlations between different groups, i.e., 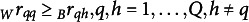. Additionally, the ultrametric property must hold for the correlations between groups. Hence, a 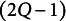 -ultrametric correlation matrix has a reduced number of parameters than a 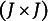 correlation matrix since its off-diagonal elements assume one of the 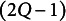 different values, i.e., 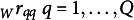, and 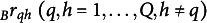. It is easy to show that a 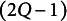 -ultrametric correlation matrix is one-to-one associated with a hierarchy of *Q* groups of MVs (Cavicchia et al., 2020, Lemma 1). In fact, as shown in Figure 1b, the first *Q* values 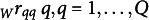, define the first-order aggregation levels of the hierarchy and represent the expected correlation within the first *Q* groups. The other 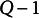 values 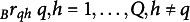, identify the remaining 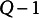 levels and represent the expected correlation between groups of MVs.

### 2.2 Exploratory sactor and disjoint factor analysis

Let 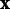 be a 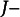 dimensional random vector with mean vector 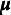 and variance–covariance matrix 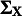 . We assume that MVs are standardized; therefore, 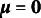 and the variance–covariance matrix 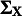 is the correlation of 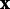 . The EFA assumes that the observed units are explained (reconstructed, generated) by the following model 
(1)

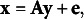

where 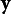 is a vector of non-observable *Q* random variables called factors, while 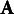 is a 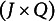 matrix of unknown parameters called *factor loadings* that identifies the statistical relations between MVs and factors, and finally 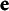 is a 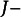 dimensional vector of non-observable random errors, whose elements are called the *specific* or *unique factors*. We shall assume that LVs and errors are centered, i.e., 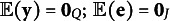 ; factors are standardized, and uncorrelated for EFA, i.e., 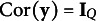 (we assume the orthogonal EFA hereinafter); and, finally, errors and factors are uncorrelated, i.e., 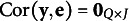 . For the Maximum Likelihood (ML) estimation, it is also assumed that 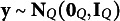 and 
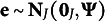
 , where 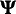 is a diagonal positive definite matrix. Given these assumptions, it can be found that 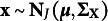 , where 
(2)

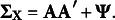



The EFA model clearly has a problem of identification of 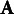 from 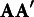 , since if 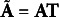 is an arbitrary orthogonal transformation of 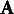 , then 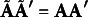 . This indeterminacy is usually solved by rotating the factor loadings to create maximum contrast between columns of 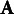 , e.g., by using varimax rotation method (Kaiser, 1958). This frequently simplifies the loading matrix and facilitates interpretation. In this spirit, Vichi (2017) introduced the DFA, in which the solution is constrained to have the *simplest* loading matrix 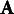 that allows each MV to be uniquely correlated with one of the *Q* LVs. Thus, 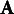 is parametrized by considering the product 
(3)

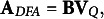

where 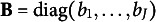 is a 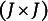 diagonal matrix and 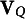 is a 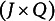 binary and row stochastic matrix. The elements of 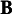 identify the levels of correlation between MVs and LVs, while 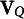 is a membership matrix indicating each MV to which LV is assigned. This parameterization induces a partition of MVs into *Q* disjoint groups and the definition of a LV for each subgroup of MVs. Therefore, the model in Eq. (2) can be written as 
(4)

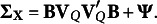



The ML estimation is obtained supposing that a random sample of 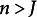 multivariate units 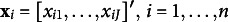 , so that 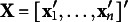 is observed; thus, the reduced log-likelihood is 
(5)

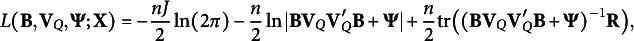

where 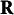 is the sample correlation matrix (equal to the sample variance–covariance matrix 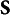 since the observed sample is standardized). It is worth noting that the maximization of 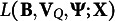 is equivalent to the minimization of the following discrepancy function with respect to 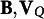 and 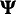



(6)

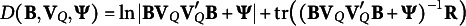

such that 
(7)

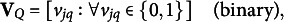



(8)

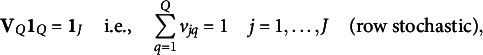



(9)

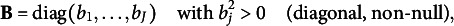



(10)

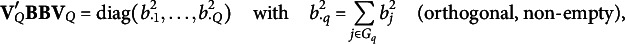

where 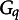 indicates the group of MVs related to the *q*th factor. DFA involves a mixed binary (in 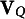 ) and continuous (in 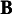 and 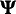 ) optimization problem, thus, a quasi-Newton method cannot be properly applied. Two coordinate descent algorithms (Zangwill, 1969) have been developed to solve it in Vichi (2017).

EFA and DFA models are scale invariant under linear transformation of the data matrix 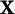 , useful for data normalization or standardization. However, DFA, differently from EFA, has the additional property to be identified (unless for a label switching), thus, no rotation is needed.

## 3 The UFA model

The UFA models hierarchical structures by providing a SF loading matrix capable of identifying the ultrametric correlation matrix describing the set of the supposed nested latent concepts. Therefore, let us formulate the mathematical form of the ultrametric correlation matrix that UFA can derive. First suppose that *Q* factors are identified according to the DFA model in Eq. (4) with the following parsimonious formulation of the loading matrix (Parsimonious DFA, PDFA) 
(11)

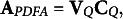

where 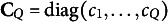 is a diagonal matrix of order *Q*. The elements of 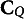 represent equal levels of correlation between each LV and the corresponding MVs. Therefore, the correlation structure of PDFA is 
(12)

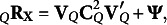

where the loadings of the MVs belonging to the same group, i.e., associated with the same LV, are equal. After a proper permutation of the rows of 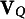 so as to have all the ones in each column of 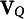 contiguous, the correlation structure in Eq. (12) has the following block diagonal form 
(13)

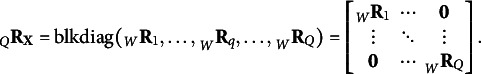

In (13), blocks represent the correlation matrices 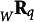 of the *q*th group of MVs related to the *q*th factor, for 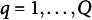 , that is 
(14)

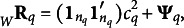

where 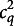 is the correlation between the *q*th factor and the 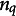 associated MVs, and 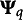 is a diagonal matrix such that 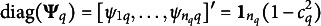 . It is worth noting that 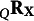, due to its block diagonal form, automatically satisfies the ultrametric conditions of Definition 1. However, for the non-orthogonal EFA, correlation between factors is hypothesized to be different from zero, i.e., 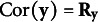 . To model the latter, an higher-order correlation structure is required, corresponding to additional “*layers*” that account for it. For the second-order correlation structure, we have 
(15)

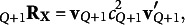

where 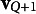 —hereinafter, we denote 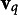 as the *q*th column of 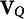 —identifies a group of variables which is obtained as the sum of two columns *h* and *q* of 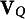 , i.e., 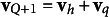 , with *h*, 
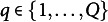
 , 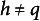 , while 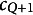 is the loading of the 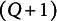 th factor corresponding to the square root of the correlation between factors *h* and *q*. The matrix formulation of Eq. (15) is 
(16)

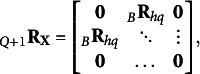

where 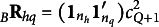. Note that the matrix obtained as 
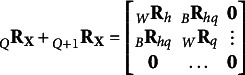
 is ultrametric, since if the *h*th and *q*th groups are aggregated it must hold that 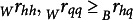 (see Section 2.1). For the additional orders of correlation structures from 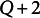 to 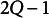 , it is supposed that the correlation not explained by the previous levels is partially explained by the successive one. Formally, the additional layers are 
(17)

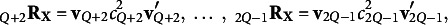

where vectors 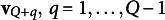 , represent the broader groups obtained by the aggregation in pairs of those from the previous levels.

The UFA model parameterizes a correlation matrix of order *J* as the sum of 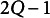 different layers as follows: 
(18)

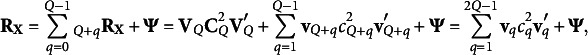

where the values 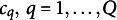 , in the diagonal of 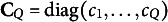 represent the loadings between the *q*th factor and the MVs in the *q*th group of the initial partition in *Q* groups, while the values 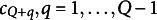 , are the loadings of the higher-order factors. It is easy to see that the right-hand side of Eq. (18), i.e., the second and third term, is, by construction, a 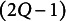 -ultrametric correlation matrix with values 
(19)

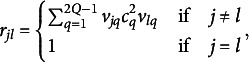

such that 
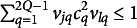
 and 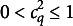 . Additionally, 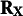 in Eq. (18) can be rewritten in a compact matrix form as follows: 
(20)

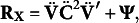

where 
(21)

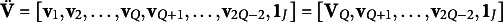

is the 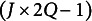 binary matrix, with the first *Q* columns of 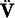 reported in 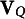 which identify the partition of the *J* MVs in *Q* groups, while the following 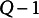 columns identify the aggregations of groups of MVs into partitions formed by 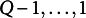 , nested groups. The last column of 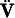 corresponds to the total aggregation of the MVs into a single class. The UFA loadings in each of the 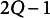 layers are stored into the matrix 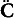 , which is given by 
(22)

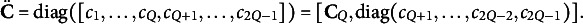

It is worth noting that the loadings 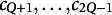 produce *cross-loadings* w.r.t. 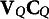 , i.e., MVs that load on two or more higher-order LVs and that induce their correlation. However, while the number of parameters in a generic non-negative correlation matrix increases in the order of 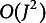 , for 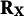 in Eq. (18), the number of parameters increases linearly in the matrix as we will see in Section 5.

Remark 1. The elements 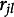 of the correlation matrix 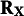 in Eq. (18) can be decomposed in two parts as follows 
(23)

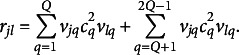

In (23), the first sum is equal to zero if MVs *j* and *l* belong to two different groups of 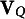 . It is worth noting that, in the first sum, at most one of the *Q* terms is non-zero (i.e., the term corresponding to the case where *j* and *l* belong to the same group), while in the second sum, which has 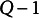 terms, more than one term may be non-zero.

Remark 2. When 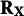 in Eq. (18) has a block diagonal form as in Eq. (13), that is, the *Q* LVs are uncorrelated, all the correlation between MVs is due to the correlation among MVs within the *Q* groups, i.e., 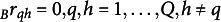. In this case, PDFA best explains the correlation in 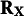 , and therefore it can be considered a special case of UFA where only 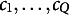 are different from zero.

### 3.1 Model properties

In this section, we illustrate the fundamental properties of the UFA model, which consist of uniqueness, internal consistency, and unidimensionality. It is thus worth recalling that a group of MVs is *internally consistent* when the corresponding factor explains them coherently, where coherence means that given any triplet 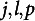 of MVs, if *j* is positively correlated (concordant) with *l* and *p*, then also the correlation between *l* and *p* is positive. Moreover, a group of (at least two) MVs is *unidimensional* if the first largest eigenvalue of their correlation matrix is greater than 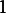 , and all the others are 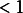 . This means that there exists a unique factor that explains most of their total variance.

Property 1 **(Uniqueness).**

The loading matrix 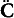 is unique, up to a permutation of columns of 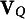 .

Proof. This is a direct consequence of DFA, which has a unique solution for the loading matrix (Vichi, 2017, Property 2). Given 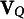 , 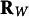 , and 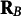 are unique and therefore also the loading matrix 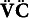 . Any transformation of 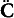 with an arbitrary orthogonal matrix 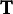 , i.e., 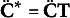 , produces a non-admissible loading matrix for UFA.

Property 2 **(Reliability).**

The matrices 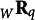 in Eq. (14) define each one a factor which is generally reliable.

Proof. The Cronbach’s alpha of 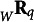 is 
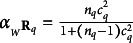
, where for 
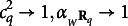
 (Brown, 1910; Spearman, 1910). It easily follows that if 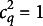 , then 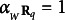.

An example of this property is as follows. Supposing that 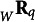 has size 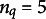 and the loading is 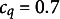 , 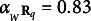, which corresponds to a *good* internal consistency (reliability, coherence). For 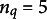 , but this time 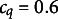 , 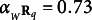, which is still an *acceptable* reliability.

Property 3 **(Unidimensionality).**

The *Q* first-order factors identified by UFA are unidimensional. Indeed, the matrices 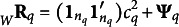 have the first largest eigenvalue equal to 
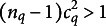
 , while all the remaining 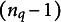 eigenvalues have value 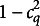 , which is smaller than 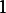 , since 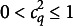 .

Proof. Denote with 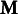 the 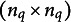 matrix of unitary elements 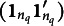 . We have that 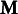 is such that 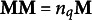 , hence 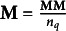 . Let 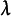 be an eigenvalue of 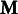 and 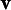 the corresponding eigenvector, then we have that 
(24)

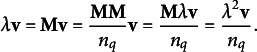

Considering that 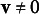 , 
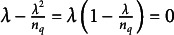
 . Therefore, either 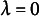 or 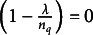 , i.e., 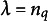 . Since, by definition, the trace of a square matrix is the sum of its diagonal elements, we have that 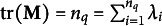 , hence it must hold that only one 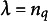 and all the other 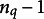 eigenvalues must be equal to 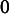 . Similarly, consider 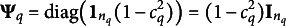 , then 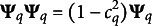 and therefore 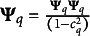 . Since 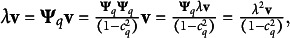 we have that either 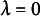 or 
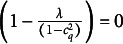
 , i.e., 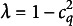 . Given that 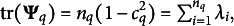 it must hold that 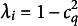 for all 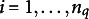 . As a consequence, the matrices 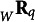, 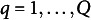 , are each one associated with a unidimensional factor, as they have only the largest eigenvalue greater than 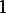 .

It is worth stressing that UFA’s structural constraints serve to limit the solution space and substantially reduce the likelihood of encountering improper solutions, such as negative variances or non-convergent estimates. Unlike traditional EFA, UFA imposes strict structural constraints on the factor loading matrix, ensuring a well-defined hierarchical and nested factor structure. These constraints effectively limit the solution space, preventing issues like Heywood cases or inflated factor loadings. As a result, UFA is less prone to the common estimation problems associated with unconstrained factor models, offering a more stable and reliable solution for analyzing data with hierarchical structures.

## 4 UFA algorithm and ML estimation

The ML estimation of UFA is performed by minimizing the following discrepancy function with respect to 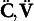 , and 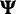



(25)

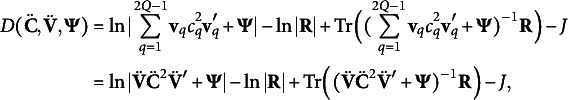

such that 
(26)

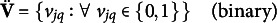



(27)

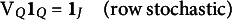



(28)

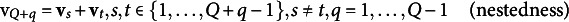



(29)

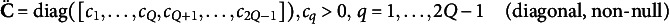



(30)

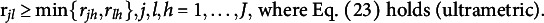



Given *Q*, the estimation of the UFA parameters is performed via a cycling block coordinate descent algorithm composed of the following four steps, which are sequentially repeated until a stopping rule is satisfied.

1. [*Initialization*] The matrix 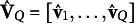 is randomly generated from a multinomial distribution with equal probabilities 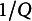 and under the constraint that the *Q* groups are not empty. The initial values for 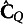 are obtained taking the square root of 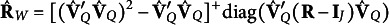 , as in Cavicchia et al. (2020). Thus, matrices 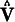 and 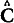 are initialized as 

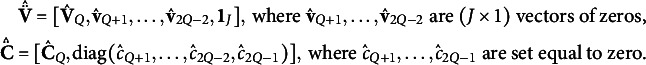

The initial 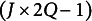 UFA loading matrix 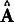 is computed as 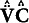 . Matrix 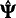 is obtained as: 
(31)

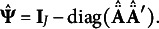

2. [*Update* 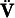 ]
**Step 1.1** [*Update* 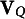 ] Matrix 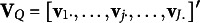 , where 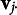 denotes the generic row *j* of 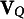 , is updated row-by-row by assigning each MV *j* to the group *q* that most decreases the discrepancy in Eq. (25). Formally, 
(32)

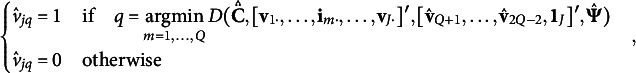

where 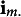 is the *m*th row of the identity matrix of order *Q*.
  Then, correlations are updated as in Cavicchia et al. (2020), i.e., 
(33)

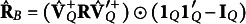



(34)

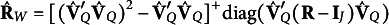

**Step 1.2** [*Update last* 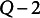 *columns of* 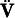 ] Given 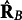 , we update the 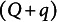 th vector 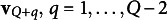 , by aggregating the two vectors 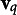 and 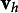 corresponding to the two factors with maximum between correlation 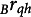, 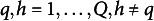 . This is the agglomerative part of the algorithm that considers the correlations between groups. Note that, if after the *q*th iteration columns *q* and *h* are aggregated, the labels of 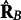 in positions *q* and *h* will be both replaced by 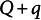 . Thus, after each iteration, we move from *Q* groups of MVs to 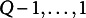 .
3. [*Update* 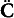 ]
**Step 2.1** [*Update* 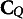 ] Given 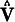 and 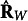 , the first *Q* values of matrix 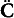 are updated as 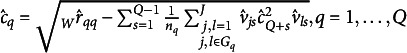.
**Step 2.2** [*Update the last* 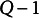 *columns of* 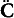 ] Given 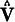 and 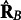 , the 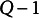 higher order levels are updated as 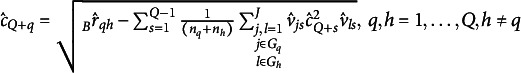.
4. [*Update* 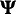 ] Given 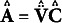 , the discrepancy function 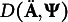 in Eq. (25) is minimized with respect to 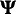 by 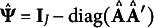 .

The last three steps are alternated repeatedly, and with each iteration, the discrepancy function decreases, or at least does not increase. If the decrease of the discrepancy is larger than an arbitrarily small positive constant, the algorithm continues to iterate; otherwise, it stops and it is considered to have converged to a solution which is at least a local minimum. To avoid the well-known sensitivity of the coordinate descent algorithms to the starting values and to increase the chance of finding the global minimum, the algorithm should be run several times starting from different initial estimates of 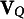 , retaining the best solution. The algorithm generally stops after a few iterations. In our simulation study reported in Section 6, this number is always less than 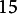 , and it can be observed that the algorithm accurately identifies the hierarchical structure in factors of MVs generated according to the UFA model.

Remark 3.

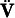
 defines a set of subgroups of MVs, described by their binary vectors, such that 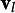 is the binary vector identifying the non-empty *l*th subgroup of 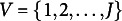 , and if 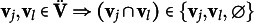 . This structure is a *J*-tree formed by *J* leaves corresponding the MVs 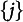 , with 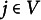 . Then, the first *Q* groups of MVs, corresponding to the first *Q* internal nodes of the tree, define a partition of *V* in *Q* disjoint groups. The successive 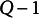 steps specify the agglomeration of the *Q* groups in 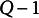 steps of group-fusions up to the root of the tree.

Remark 4. In general, there is no reason why the optimal estimation of UFA should include the PDFA solution at the *Q*th level. However, if one wants to improve the algorithm’s computational efficiency, the estimates in Steps 1.1 and 2.1 can be replaced by the PDFA solution at convergence. This avoids computing Eqs. (32), (33), and (34) at every iteration. Therefore, we define a new constrained algorithm for UFA that is forced to include the optimal solution of PDFA for the given *Q*.

The following example is provided to help the reader better follow the UFA modeling.

**Figure 2:**
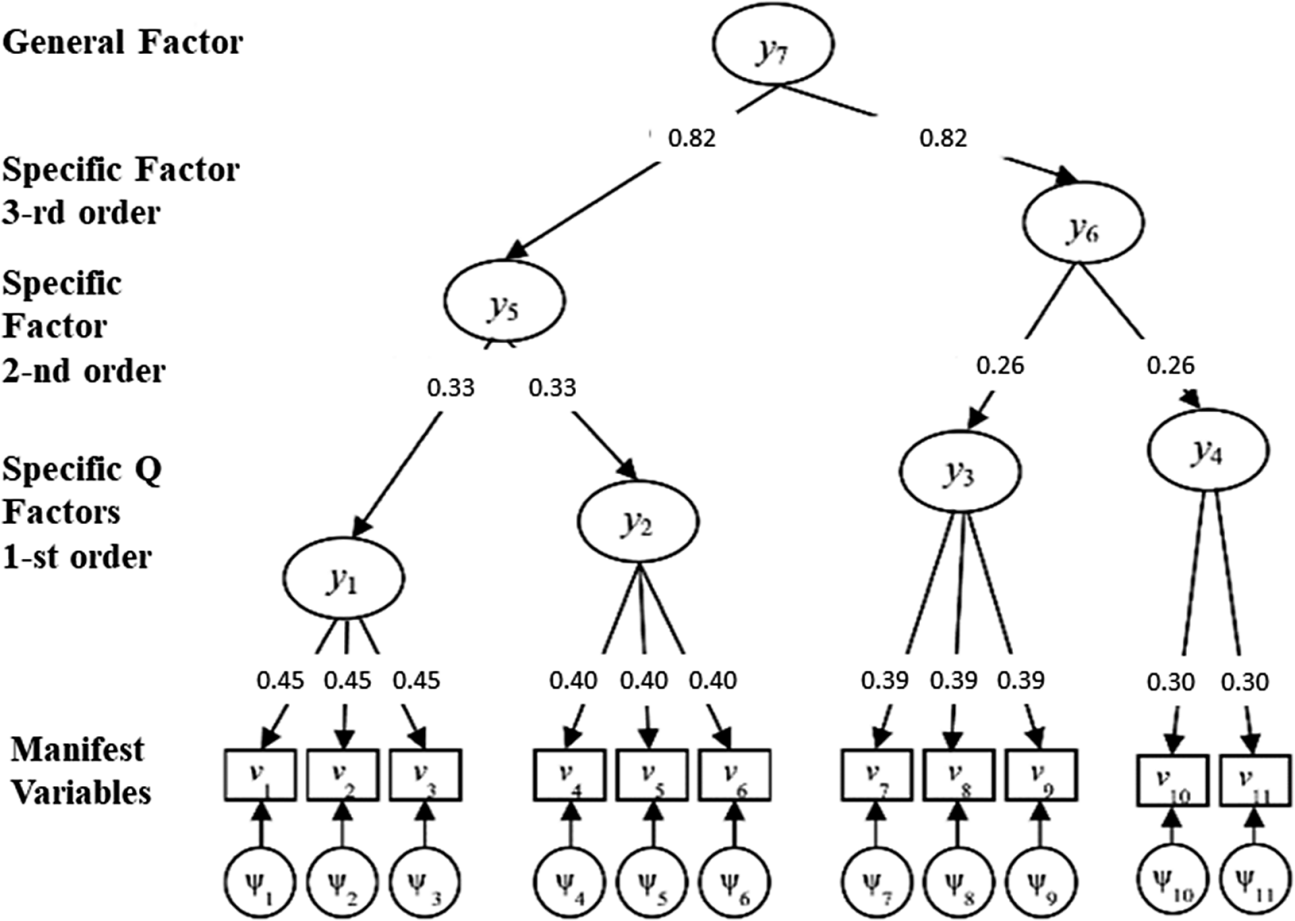
Path diagram for the given example.

Example. We apply UFA to the set of eleven MVs in the example of Figure 1. In the correlation matrix in Figure 1a, 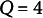 groups of highly correlated variables are clearly visible: 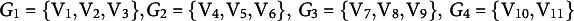 . The specific *Q* first-order factors are characterized by loadings 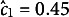 , 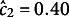 , 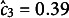 , and 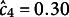 , as reported in Fig 2. The loadings are computed considering the within correlations in Figure 1a as reported in **Step 2.1** of the algorithm and detailed in *Remark* 1. The membership matrix for this partition is identified by the first four columns 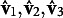 , and 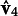 of matrix 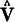 . The within and between correlations of groups/factors are given by 

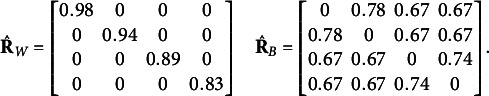

Let us now consider the hierarchical structure of factors—from the most specific ones (first-order) to the most general, through second- and third-order SFs—as shown in the higher layers of the path diagram in Figure 2. The second-order SF (2nd layer) agglomerates the first two groups of MVs since their correlation is the highest in 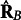 (equal to 0.78). Therefore, 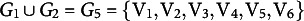 , where this aggregation is identified by the vector 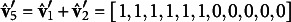 representing the fifth column of matrix 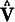 . The corresponding loading is 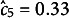 . Having merged these two groups, the third-order SF (3-rd layer) lumps together groups 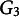 and 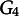 (the correlation equals 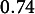 ), i.e., 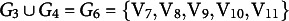 , obtaining the additional vector 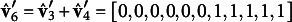 and 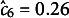 . The last aggregation corresponds to 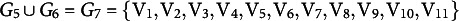 , where 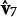 is the unitary vector of dimension eleven and 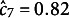 . All higher-order loadings can be found by considering the between correlations in 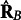 , as reported in **Step 2.2**. As a result, the UFA membership matrix and the corresponding loading matrix are given by 

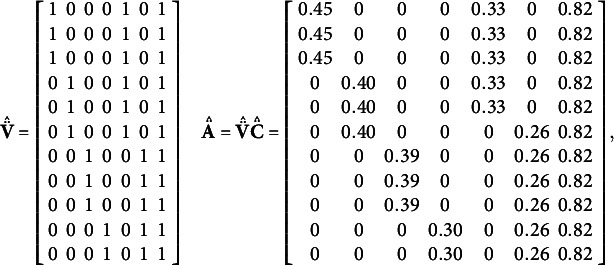

where the first *Q* columns of 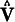 correspond to 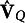 . Finally, 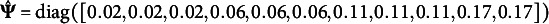 .

## 5 Model selection and choice of *Q*

In this section, we discuss the choice of the optimal UFA model, among a set of candidates that consider different values of *Q*. The optimal model is the one that strikes the best balance between complexity and ability to accurately fit the observed data. Through this careful selection, we determine the appropriate number of factors *Q* to retain, ensuring both efficiency and effectiveness in our analysis. To assess the complexity of the model, we start from noticing that the population correlation matrix has 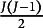 parameters to be estimated (the correlations on the diagonal are always equal to 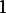 ) in terms of 
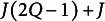
 unknown parameters in 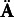 and 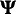 . However, note that the first *Q* columns of 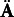 have only one loading different from 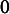 for each row. Thus, we have 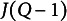 constraints in 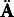 . Moreover, we have other *Q* constraints on 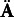 to ensure that each of the first *Q* columns of 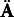 is not empty. Finally, we suppose that all MVs loading on the same factor have the same coefficient 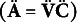 , therefore we do not have to estimate *J* coefficients but only *Q*; this corresponds to have 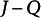 additional constraints on 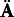 . This latter condition also holds for the last 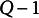 columns of 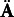 : we do not have to estimate 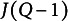 coefficients but only 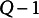 and thus we have additional 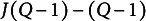 constraints on 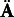 . Since we have the same coefficient for all MVs loading onto the same factor, also for the diagonal of 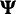 we do not have to estimate *J* parameters but *Q*, and therefore there are 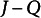 constraints on 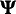 . Hence, the effective number of unknown parameters in UFA is 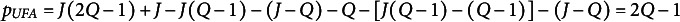 . Note that, due to the factorization of 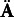 , 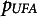 does not depend on *J*. The degrees of freedom of the model are therefore 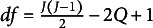 .

Two popular model selection criteria are the Akaike Information Criterion (AIC, Akaike, 1998) and the Bayesian Information Criterion (BIC, Schwarz, 1978), which can be easily estimated for the UFA model, given Eq. (25) and 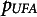 . Among a set of candidate models (i.e., different values of *Q*), the preferred is the one for which AIC and BIC are minima. Alternatively, a goodness of fit index frequently used to address this task is the Adjusted Goodness of Fit Index (AGFI, Mulaik et al., 1989), which is computed as follows: 
(35)

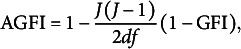

where GFI is the Goodness of Fit Index (GFI, Jöreskog, 1981) obtained as 
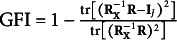
 . This selection criterion chooses *Q* such that AGFI is maximized.

It is worth stressing that even if these criteria are well-recognized and commonly used, AIC and BIC can be more helpful for model comparison (i.e., selecting the optimal *Q*), while AGFI is more appropriate for evaluating model fit once the number of first-order factors *Q* has been chosen. Furthermore, in practice, combining multiple methods—both statistical and conceptual—typically leads to the most robust factor solution.

Remark 5. Note that when performing non-parsimonious DFA, the number of parameters to be estimated is 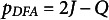 (Vichi, 2017), whereas for PDFA, this number is 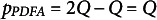 . It can be immediately seen that 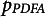 corresponds to UFA without considering the 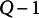 parameters of the factorial hierarchy. In fact, 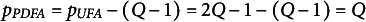 .

## 6 Simulation study

The proposed model is evaluated through an extended simulation study that considers twelve different scenarios and involves 3,000 data samples, generated according to the following procedure. First, the matrix 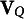 is randomly generated. Then, a random binary *J*-tree is generated starting from 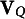 and including 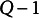 random agglomerations of subsets (couples). The matrix 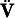 is identified by this *J*-tree. As a second step, a diagonal matrix 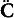 with non-increasing values in 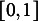 is generated, and the loading matrix 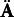 is obtained as 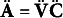 . The matrix 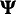 is then computed accordingly using Eq. (31). The correlation matrix is obtained as 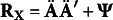 , and it is 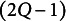 -ultrametric by construction. Finally, the observed correlation matrix 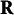 is obtained adding to 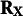 a uniform error 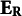 in 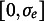 , which is symmetrized. The psd of 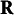 has been verified: if this property is not satisfied, we add the absolute value of the smallest eigenvalue of 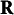 to its main diagonal, so that the resulting matrix is positive semi-definite (Cailliez, 1983).

**Figure 3:**
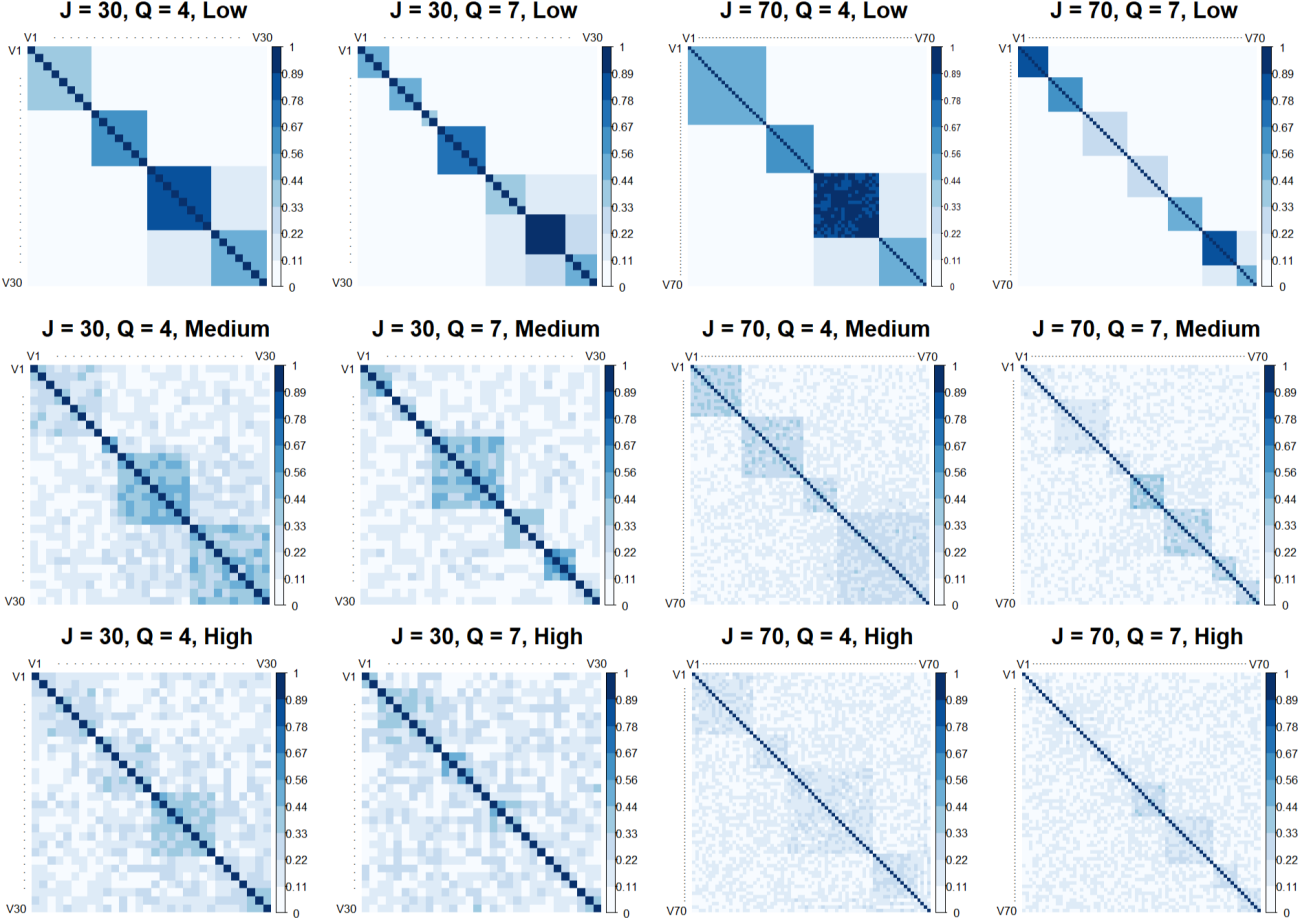
Examples of simulated correlation matrices with different levels of error, *J* and *Q*.

Twelve different scenarios have been taken into account to test the proposed model. We considered 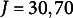 MVs, 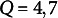 factors and three error levels 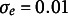 , 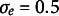 , and 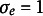 . In Figure 3, examples of simulated correlation matrices for each scenario are illustrated. The groups and their hierarchical structure are clearly visible in the first row of Figure 3. In the four plots of the second row, corresponding to the medium error level, the hierarchical structure of the *Q* first-order factors is still quite visible while the one for higher-order aggregations tends to disappear. Finally, for the high error case (third row of Figure 3), the whole hierarchical structure among both MVs and LVs almost disappears.

### 6.1 Random starts assessment

UFA does not guarantee the identification of the global optimal solution, which is expected since the partitioning problem is known to be NP-hard (Křivánek & Morávek, 1986). To increase the chance of find the global optimum, the *multistart* technique is used and, in this subsection, it is discussed how the number of random starts is chosen. To address this, the UFA algorithm is run 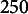 times with a high error level and 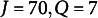 , using different numbers of *randstarts* values: 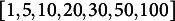 . For each case, the percentage of local minima is recorded.

From the results in Table 1, the number of random starts for the simulation study is set equal to 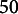 .

**Table 1:** Local minima occurrences (%)

*randstarts*	1	5	10	20	30	50	100
% Local minima	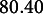	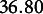	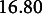	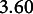	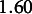	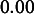	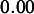

### 6.2 Model performance and comparison with competing procedures

The proposed model is evaluated according to the Adjusted Rand Index (ARI, Hubert & Arabie, 1985), which compares the generated *J*-tree with the estimated one. First, the ARI between the true and estimated matrices 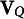 for the first *Q* aggregations is computed. Then, an ARI is computed for each of the remaining 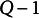 aggregations levels. For each 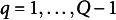 , the ARI is computed between the two factors aggregated by the algorithm at level 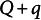 and the two generated ones. As a result, the generated *J*-tree is optimally recovered if the overall ARI equals to *Q*, since it is obtained as the sum of one ARI for the first *Q* levels and 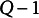 ARIs for the factors’ hierarchy. Finally, this value is rescaled, dividing it by *Q*, to range in 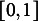 . We will denote with ARI 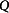 the ARI referring to the first *Q* levels and with ARI 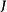 the overall ARI for the *J*-tree. Furthermore, the fit of the 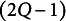 -ultrametric correlation matrix resulting from UFA to the generated 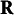 is evaluated through the AGFI. The discrepancy between the generated loading matrix, denoted with 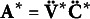 and the estimated one 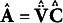 is evaluated considering the Root Mean Squared Error (RMSE) defined as 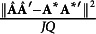 since the loading matrix is not unique.

UFA performance is compared with the ones of several well-established factor rotation methods, such as geomin (geo), promax (pro), and quartimax (qua), as well as with the *prenet* penalization method for factor analysis proposed by Hirose & Terada (2023). Geomin, introduced by Yates (1987), minimizes the complexity of factor loadings by reducing the number of near-zero values. Promax, a widely used oblique rotation method proposed by Hendrickson & White (1964), allows factors to correlate, thereby enhancing the interpretability of the factor solution. Quartimax, introduced by Neuhaus & Wrigley (1954), seeks to simplify the rows of the factor loading matrix by maximizing the variance of squared loadings across variables. The Sparse and Simple Structure (SSS) estimation method developed by Hirose & Terada (2023) employs the *prenet* penalization to achieve a factor solution that is both sparse and interpretable. This estimation requires tuning two parameters, denoted by 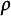 and 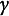 , and has been carried out to maximize AGFI. By comparing these techniques with UFA, which offers an innovative approach to factor analysis, we aim to highlight its strengths and potential advantages in achieving more interpretable and accurate factor solutions when data exhibit a hierarchical structure.

Simulation results, obtained considering 50 random starts for all cases, are shown in Table 2. For each scenario, 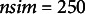 data matrices have been generated and the reported performance measures correspond to the average over the 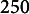 samples.

**Table 2:** Simulation study results

		Low error				Medium error				High error			
	*J*	30		70		30		70		30		70	
	*Q*	4	7	4	7	4	7	4	7	4	7	4	7
	UFA	1.00	1.00	1.00	1.00	1.00	0.98	1.00	1.00	0.97	0.72	1.00	0.93
	SSS	0.66	0.78	0.65	0.77	0.63	0.68	0.65	0.72	0.41	0.19	0.65	0.72
Mean ARI 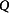	pro	0.66	0.78	0.65	0.77	0.66	0.78	0.65	0.77	0.66	0.77	0.66	0.77
	geo	0.66	0.78	0.65	0.77	0.66	0.77	0.65	0.77	0.65	0.76	0.66	0.76
	qua	0.66	0.78	0.65	0.77	0.66	0.78	0.65	0.77	0.65	0.77	0.66	0.76
	UFA	100.00	100.00	100.00	100.00	99.60	86.80	100.00	100.00	79.60	3.20	99.60	34.00
	SSS	0.00	0.00	0.00	0.00	0.00	0.00	0.00	0.00	0.00	0.00	0.00	0.00
% ARI 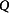 = 1	pro	0.00	0.00	0.00	0.00	0.00	0.00	0.00	0.00	0.00	0.00	0.00	0.00
	geo	0.00	0.00	0.00	0.00	0.00	0.00	0.00	0.00	0.00	0.00	0.00	0.00
	qua	0.00	0.00	0.00	0.00	0.00	0.00	0.00	0.00	0.00	0.00	0.00	0.00
Mean ARI 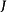	UFA	1.00	0.98	1.00	0.98	1.00	0.85	1.00	0.93	0.96	0.70	1.00	0.83
% ARI 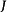 = 1	UFA	100.00	86.00	100.00	86.40	97.20	4.80	100.00	38.40	62.40	0.00	98.80	3.60
	UFA	1.00	1.00	0.99	1.00	0.78	0.78	0.79	0.79	0.77	0.77	0.78	0.79
	SSS	0.86	0.91	0.83	0.89	0.51	0.54	0.69	0.69	0.43	0.46	0.67	0.62
AGFI	pro	0.98	0.97	0.99	0.98	0.64	0.45	0.74	0.71	0.63	0.44	0.74	0.70
	geo	0.98	0.97	0.99	0.98	0.65	0.51	0.75	0.72	0.65	0.52	0.74	0.73
	qua	0.98	0.97	0.99	0.98	0.65	0.52	0.75	0.72	0.65	0.53	0.74	0.73
	UFA	0.00	0.00	0.00	0.00	0.16	0.08	0.60	0.19	0.28	0.14	0.88	0.30
	SSS	1.22	0.44	2.72	0.97	1.08	0.39	2.31	0.81	1.48	0.40	7.86	0.82
RMSE 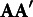	pro	1.26	0.44	2.95	1.03	1.03	0.40	2.23	0.79	1.03	0.37	2.24	0.79
	geo	1.25	0.44	2.93	1.02	0.98	0.36	2.18	0.77	0.98	0.34	2.16	0.77
	qua	1.26	0.44	2.95	1.03	0.98	0.36	2.20	0.77	0.98	0.33	2.17	0.77

Under the low error condition, our method demonstrates best performance across all evaluated metrics. Specifically, the generated *J*-tree is always perfectly identified for 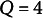 , while the percentage of times this happens is around 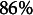 for 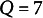 (see Table 2). Moreover, the AGFI is always greater than or equal to 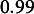 and the RMSE for the loadings reaches 0. In contrast, the competing procedures poorly perform in recovering the variables structure in *Q* groups: the ARI 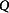 always ranges between 0.65 and 0.78 and it is never equal to 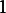 . Additionally, the high values for the RMSE of the loading matrix indicate a sub-optimal recovery of the generated loadings across all scenarios. This first part of the simulation study indicates that the proposed methodology and the given algorithm do not fail to identify the correct generated ultrametric correlation matrix when its ultrametricity is clearly visible, as shown in the first row plots of Figure 3. In comparison, the competing procedures fail to achieve an accurate identification under the same conditions.

For the medium error level scenarios, the UFA algorithm achieves best performance in terms of ARI 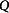 , while the recovery of the higher-order hierarchies starts worsening. Indeed, in the plots of Figure 3 corresponding to the medium error level, the structure in *Q* factors is still visible while the higher-order correlations start deviating from ultrametricity. For instance, for 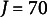 and 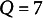 , the 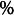 ARI 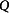 = 1 and the % ARI 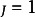 are 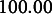 and 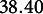 , respectively. Also the model-data fit in terms of RMSE and AGFI worsens, but the fit is still better than the competing procedures.

Under the high error condition, as expected, the UFA performance also in terms of ARI 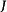 worsens because the ultrametricity tends to be masked by the error, as can be seen in the last row of Figure 3. The true *J*-tree is never identified for 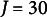 and 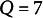 (under the same scenario, the % ARI 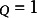 is 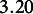 ). However, also under these scenarios, UFA outperforms the competing procedures, especially in terms of ARI 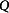 and RMSE.

**Figure 4:**
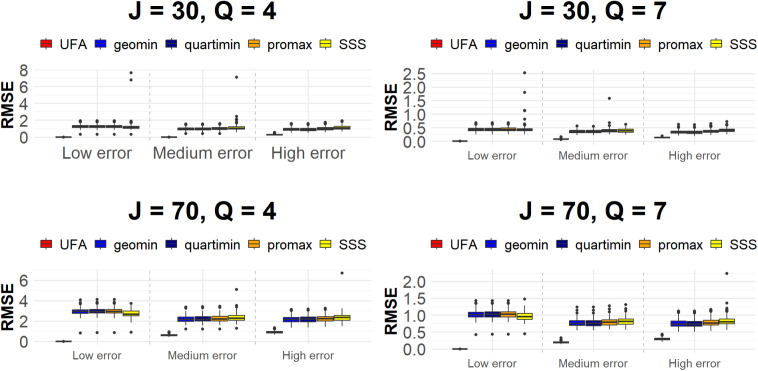
RMSE of factor loadings for UFA and the competing procedures.

As illustrated in Figure 4, UFA shows the lowest RMSE across all scenarios, with its interquartile range being very tight, indicating both a low error and consistent performance across simulations. In contrast, the competing methods exhibit higher median RMSE values and wider interquartile ranges, indicating poorer performance and higher variability. These results underscore the efficacy and accuracy of UFA in capturing hierarchical relationships in data, making it a reliable choice for this kind of scenarios, compared to traditional methods.

To summarize, simulation results collectively suggest that the proposed algorithm not only achieves high accuracy in identifying the hierarchical structure of the generated data and finds its best ultrametric reconstruction, but also exhibits stability and reliability in its performance. The effectiveness of the algorithm against the benchmark criteria and competing procedures suggests its potential utility in applications in which phenomena that can be modeled by a hierarchical latent structures are studied.

## 7 Application

The proposed model is applied to the data employed by Cattell (1971) for its factorial study on intelligence, in which the author investigates his theory of crystallized and fluid intelligence factors. Crystallized intelligence involves the ability to use learned knowledge and experience. It involves understanding facts, solving problems based on past experiences, and possessing a rich vocabulary, and it tends to improve with age. On the other hand, fluid intelligence represents the ability to solve novel problems, independent of any knowledge from the past. It includes skills like pattern recognition, abstract reasoning, and problem-solving in new situations. Fluid intelligence is thought to peak in early adulthood and then gradually decline. Cattell’s study used data from 277 8th grade children, focusing on their abilities across different dimensions. These dimensions were measured through various tests:

- Thurstone Primaries: four areas (Verbal, Spatial, Reasoning, Number) were each measured by two different tests, resulting in eight measures (V1, V2, S1, S2, R1, R2, N1, N2). These areas represent different cognitive abilities as identified by Thurstone (1938), another key figure in the study of human intelligence.
- Culture Fair Intelligence Tests: developed by the Institute of Personality and Ability Testing (IPAT), these tests aim to measure intelligence in a way that minimizes cultural and educational biases. The tests include Series, Classification, Matrices, and Topology, each providing a score (IS, IC, IM, IT).

**Figure 5:**
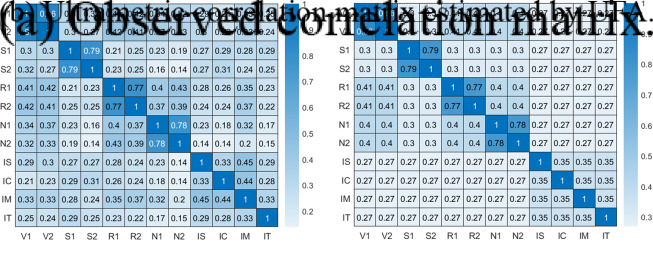
Observed and estimated correlation matrices of Cattell’s data.

The non-negative correlations among the 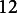 considered MVs are shown in Figure 5a. These high correlations suggest the need to explore how the various aspects of intelligence interrelate and potentially validate or refine Cattell’s theory of crystallized and fluid intelligence.

**Figure 6:**
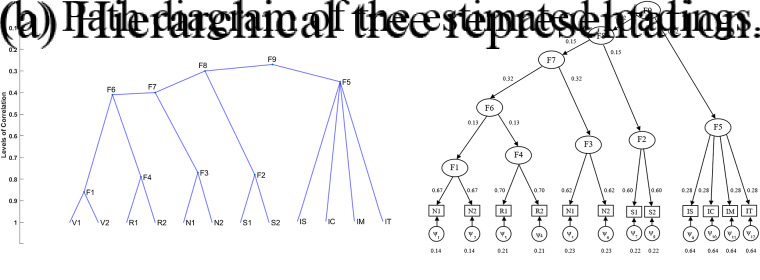
Hierarchical tree and loading structure estimated by UFA on Cattell’s data.

The UFA algorithm has been run considering 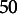 random starts and it has found the expected partition in five first-order factors (Verbal, Spatial, Reasoning, Number, and IPAT). It is worth noting that 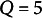 is the best choice for the number of parameters also according to the AGFI, AIC, and BIC. The unidimensionality of the identified latent dimensions is ensured by the variance of the second factor equal to 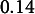 , 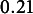 , 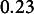 , 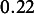 , and 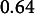 , respectively. The Cronbach’s alpha values computed for each factor align with Cattell’s hypothesis: they are greater than 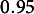 for F1, F2, F3, F4, while equal to 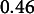 for F5. As it is possible to observe comparing the two matrices in Figure 5, the proposed model is able to well reconstruct the correlation matrix of the MVs, simultaneously identifying its hierarchical structure. The latter is depicted in Figure 6a: it can be noticed that the second last aggregation (F5–F8) characterizes the fluid ability and crystallized ability factors hypothesized by Cattell.

Moreover, the last factor aggregated to build F8 is the one related to the Spatial ability, the one for which Cattell hypothesized the strongest relationship with the IPAT MVs (F5). This is confirmed by the lowest Cronbach’s alpha value for F8 ( 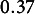 ) compared to the values for F6 and F7 ( 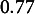 and 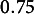 , respectively); indeed, Cattell hypothesized an high correlation between Verbal, Reason, and Number. A general concept defining Mental Abilities is also defined (F9), its reliability is ensured by a Cronbach’s alpha value equal to 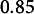 . The estimated loadings are graphically shown in Figure 6b.

Note that the estimated matrix represented in Figure 5b is ultrametric even if the within deviance of the IPAT group ( 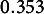 ) is higher than, for instance, the between deviance of the Verbal and Spatial groups ( 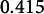 ). In fact, the ultrametric condition defined in Section 2.1 imposes that the within deviance of the IPAT group must be greater than or equal to the deviance between IPAT and the other four groups (i.e., 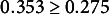 ) and not greater than or equal to any of the between deviances in the correlation matrix.

UFA results on Cattell data represent a great contribution to the state of the art in Cattell’s factorial study on intelligence. UFA does a step forward with respect to both first-order and second-order CFA that Rindskopf & Rose (1988) fitted to these data. It recognizes the five factors despite the fully exploratory approach and it also accounts for intercorrelations among them, going beyond the construction of just two second-order factors (with a correlation of 0.78). UFA model addresses the unexplained correlation among factors building a hierarchy of Specific LVs. UFA model has been compared with the two CFAs in terms of AIC and BIC (Table 3). As it is possible to observe, the lowest values correspond to UFA, reflecting the better modeling of the underlying factors’ correlation that characterize Cattell’s data. The goodness of the fit of the estimated UFA model is further confirmed by an AGFI equal to 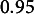 .

**Table 3:** AIC and BIC values for UFA model and for the two CFAs applied to Cattell data

Models	AIC	BIC
First-order CFA	7,890.62	8,013.84
Second-order CFA	7,887.01	7,995.73
Ultrametric factor analysis	2,289.31	2,340.04

The results obtained in this application suggest that an UFA can be the proper choice in all psychometric applications in which the researcher is interested in evaluating an hypothesized hierarchy of latent concepts and seeks to define and characterize broader dimensions of the phenomenon under study.

## 8 Conclusions

In the realm of psychometric studies, hierarchical concepts are pivotal for uncovering and comprehending fundamental phenomena, such as intelligence. In the literature, hierarchical models based on sequential procedures of EFA followed by oblique rotations have been proposed to arrange various facets or subcomponents of a construct into a hierarchical structure, showcasing their interconnectedness and offering an in-depth comprehension of complex phenomena. Additional models widely employed in psychometric studies to assess the compatibility of theoretical hierarchical factorial structures with observed data are CFA and SEM. These statistical tools help confirm that the used models accurately reflect underlying constructs, offering a reliable framework for analyzing intricate data. These models are widely used across various psychometric domains, with the Five-Factor Model of personality serving as just one example. This model categorizes personality traits into five overarching factors, each subdivided into lower-level facets that depict specific aspects of these traits.

In this article, we introduce a novel methodology called UFA. This approach expands upon the traditional EFA model with the aim of identifying hierarchical structures within factors that represent latent concepts through an exploratory and simultaneous approach, as opposed to confirmatory or sequential procedures based on EFA and oblique rotations. UFA proves particularly valuable in studying complex phenomena that exhibit multiple levels of abstraction and interconnected dimensions. The proposal can be fully exploratory, meaning that no prior assumptions are made regarding the relationships among MVs, first-order factors, and higher-order factors. However, it also offers the flexibility to fix some or all of these relationships in a partially or fully confirmatory approach. UFA involves a mathematical formalization of the hierarchical relationships among variables, which enables a synthesized representation of latent concepts associated with complex and multidimensional phenomena. By extending EFA to hierarchical structures, this methodology empowers researchers to delve into the underlying structure of complex phenomena with several facets by uncovering factors and their hierarchical relationships in a simultaneous approach.

Furthermore, the new methodology addresses several crucial properties essential for constructing a hierarchy of factors. These properties include internal consistency, reliability, unidimensionality, uniqueness, and content validity. Ensuring that these properties are met guarantees that the constructed hierarchical model accurately represents the underlying constructs and provide a comprehensive framework for analyzing complex data. Simulation study results showcased the superiority of the UFA method over traditional factor analysis techniques, especially when dealing with data that have a nested or hierarchical factor structure. While conventional methods often fail to effectively capture the complexity of such data, leading to sub-optimal factor solutions, UFA excels in identifying and modeling these intricate relationships, resulting in more accurate and interpretable factor structures. In summary, the UFA methodology presents a novel approach to investigate hierarchical concepts in psychometric studies and other disciplines where latent factors need to be constructed. This methodology offers insights into the multidimensional nature of latent constructs and facilitates a comprehensive understanding of complex phenomena. It enhances our ability to analyze data and extract meaningful information from hierarchical structures, contributing to advancements in various research fields.

Further developments of UFA include relaxing the non-negativity assumption on the correlation matrix, which, while realistic in many applications—especially in psychometric studies—could be too restrictive in other fields. Additionally, when the phenomenon under study does not show a hierarchical structure, the ultrametricity assumption could also be restrictive, and an hypothesis test to determine how far the data are from this condition could be useful for discriminating the applicability of the proposal.

## Data Availability

The MATLAB source code of UFA is openly available at https://github.com/AuthorsSubmission/UFA.
